# Environmental stress and reproduction in *Drosophila melanogaster*: starvation resistance, ovariole numbers and early age egg production

**DOI:** 10.1186/1471-2148-6-57

**Published:** 2006-07-18

**Authors:** Marta L Wayne, Usha Soundararajan, Lawrence G Harshman

**Affiliations:** 1 Department of Zoology, University of Florida, Gainesville, FL32611, USA; 2 School of Biological Sciences, University of Nebraska-Lincoln, Lincoln, NE 68588, USA

## Abstract

**Background:**

The Y model of resource allocation predicts a tradeoff between reproduction and survival. Environmental stress could affect a tradeoff between reproduction and survival, but the physiological mechanisms underlying environmental mediation of the tradeoff are largely unknown. One example is the tradeoff between starvation resistance and early fecundity. One goal of the present study was to determine if reduced early age fecundity was indeed a robust indirect response to selection for starvation resistance, by investigation of a set of *D. melanogaster *starvation selected lines which had not previously been characterized for age specific egg production. Another goal of the present study was to investigate a possible relationship between ovariole number and starvation resistance. Ovariole number is correlated with maximum daily fecundity in outbred *D. melanogaster*. Thus, one might expect that a negative genetic correlation between starvation resistance and early fecundity would be accompanied by a decrease in ovariole number.

**Results:**

Selection for early age female starvation resistance favored survival under food deprivation conditions apparently at the expense of early age egg production. The total number of eggs produced by females from selected and control lines was approximately the same for the first 26 days of life, but the timing of egg production differed such that selected females produced fewer eggs early in adult life. Females from lines selected for female starvation resistance exhibited a greater number of ovarioles than did unselected lines. Moreover, maternal starvation resulted in progeny with a greater number of ovarioles in both selected and unselected lines.

**Conclusion:**

Reduced early age egg production is a robust response to laboratory selection for starvation survival. Ovariole numbers increased in response to selection for female starvation resistance indicating that ovariole number does not account for reduced early age egg production. Further, ovariole number increased in a parallel response to maternal starvation, suggesting an evolutionary association between maternal environment and the reproductive system of female progeny.

## Background

Differential allocation of resources can give rise to tradeoffs that are manifest as negative associations between traits [1,2]. Tradeoffs presumably exert a central role in life history evolution given that energy-limited organisms cannot simultaneously maximize all components of fitness. For an insect with ephemeral food sources, such as *D. melanogaster*, starvation resistance is a complex trait which is quite likely to interact with canonical life history traits such as fecundity [3,4]. In order to understand the evolutionary response of this (and other) species to food deprivation, it is important to investigate the relationship between resistance to food deprivation and life history traits.

Ovariole number variation is related to ecological conditions. Among related species of fruit flies, a greater number of ovarioles is correlated with "niche breadth" measured as the number of different kinds of fruits used by ovipositing females [5-7]. It is not clear that the fresh fruit resource of these flies is comparable to the rotting fruit habitat of *D. melanogaster*, but the main point is that there is an association between ovariole number and environmental variation. Another line of evidence for the importance of ecological variation is that *D. melanogaster *ovariole number varies with latitude on three continents: a Europe-Africa transect [8,9]; North America [10]; and Australia [11]. Such replication of clines strongly implies the action of selection in the creation and maintenance of the clines, as argued for an isozyme cline [12] or quantitative traits such as body size [13]. These clines in ovariole number might be associated with differences in temperature, duration of reproductive period, the seasonal pattern of food availability and geographic variation in over-wintering conditions. The evidence from both between and within species suggests that there may be a relationship between environmental variation and ovariole number.

Artificial selection in the laboratory is one approach to investigate the relationship between life history and stress resistance traits. In particular, indirect responses to artificial selection can reveal sets of correlations among traits some of which may suggest underlying mechanisms [14-17]. Starvation resistant lines have been shown to have a relatively long development time [18,19], and most relevant to the present study, starvation selected-lines have been observed to be genetically correlated with relatively reduced early age fecundity [20]. The latter association does not prove that the traits are linked, but it does suggest precedence for some of the trait relationships investigated here.

In the present study, we examined the relationship between starvation resistance, ovariole number and fecundity in lines of *D. melanogaster *that were artificially selected for resistance to female starvation. Our working hypothesis was that females selected for starvation resistance would exhibit decreased egg production at the age they were selected, presumably as a change in allocation from the ovary to the body, possibly by reducing ovariole number. Starvation resistance was negatively genetically correlated with reduced early age fecundity. However, total egg production for the first 26 days of life was not different between selected and control lines. Ovariole number increased in response to selection for starvation resistance. This increase cannot be explained by a change in linear body size between selected and unselected lines. To further investigate the unforeseen positiveassociation between starvation resistance and ovariole number, we studied the plastic and phenotypic relationships between starvation and ovariole number. Maternal starvation resulted in an increase in progeny ovariole number indicating an integral relationship between maternal food deprivation and the state of the ovary in the next generation.

## Results

### Ovariole number

**Figure 1:**
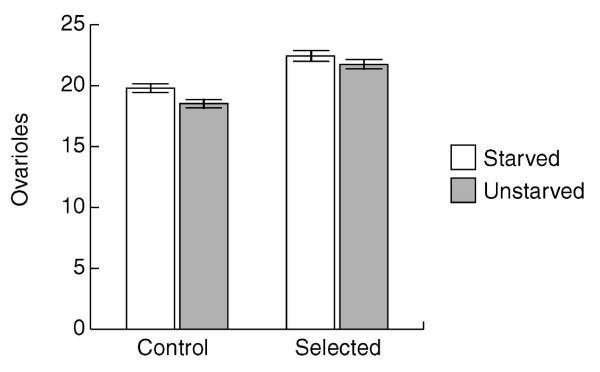
Grand average of sum of ovarioles for both ovaries, for selected (Si, right) and control (W, left) lines. The grand average was the overall mean for the five selected and five control lines; the standard error is based on the number of replicate selection and control lines. The experimental treatment was maternal starvation for either 28 hours or 32 hours (starved, shaded bars) compared to no maternal starvation (unstarved, open bars). The progeny of these females were used for ovariole number determination. The females ("dams") subjected to the experimental treatment were two generations removed from the selection regime as described in the *Ovariole number *section of the Methods or in the Results section. Significant differences between linetypes, but not between line (linetypes), are the same if flies are zero, one or two generations removed from selection.

Based on a comparison of progeny from unstarved mothers, there were a greater number of ovarioles in selected line females than in control line females (Figure 1; *P *< 0.001). Figure 1 presents the grand average number of the sum of ovarioles from both ovaries ± SE for the control and selected lines when the latter were two generations removed from the selection experiment regime. The standard errors were derived from the individual line means within line type (selected or control). The mean sum of ovarioles per female for the five control lines was 31.80, 36.60, 32.10, 34.20, and 35.60; and the mean sum of ovarioles per female for the five selected lines was 41.90, 39.70, 40.90, 44.40, and 38.90 (Table 4). Selected lines have significantly more ovarioles than control lines, whether the dams were zero, one, or two generations removed from the selective regime (mean of control lines vs. mean of selected lines: 34.48 vs. 42.17, 38.46 vs. 43.00, 38.39 vs. 44.15; Tables 1, 2, and 3, respectively). Dams refers to females used to produce daughters for ovariole number determination. Zero generations removed from the selective regime is the case in which the dams were taken from selected lines. One generation removed from the selective regime refers to the case in which the selection was relaxed for one generation in the dams of daughters used for ovariole number determination. Two generations removed from the selection regime refers to the case in which selection was relaxed for two generations in the dams of daughters used for ovariole number determination.

**Table 1:** Analysis of variance for sum of ovarioles from both ovaries (parents taken directly from selection regime). Comparison of selected *vs*. control lines when the dams of daughters for ovariole number determination was directly from the selected and control lines. Treatment refers to starved or unstarved mothers ("dams" as described in the *Ovariole number *section of the Methods or Results). Analysis uses a two way mixed model ANOVA, with Linetype (selected vs. control) and Treatment (starved or not starved) as fixed main effects. Linetype *Treatment is also fixed. Line refers to the five replicate lines in each linetype. Line is nested within Linetype and is random. Line (line type) refers to variation within selected or control lines. * =*P *≤ 0.05; ** =*P *< 0.01; *** =*P *< 0.001

Source	Df	Estimated mean squares	Variance component
Line type (selected vs. control lines)	1	2956.81***	(fixed)
	1	102.25**	(fixed)
Line type * treatment	1	17.41	(fixed)
Line (line type)	8	76.54***	3.051
Error	188	15.53	15.527

**Table 2:** Analysis of variance for sum of ovarioles from both ovaries (one generation removed from selection as described in *Ovariole number *section of the Methods or Results). Comparison of selected *vs*. control lines, using a one-way mixed model ANOVA, with line type (control or selected) as a fixed main effect and line nested within linetype as a random effect. Line (line type) refers to the five replicate lines in each line type. * =*P *≤ 0.05; ** =*P *< 0.01; *** =*P *< 0.001

Source	Df	Estimated mean squares	Variance component
Line type (selected vs. control lines)	1	1027.56*	(fixed)
Line (line type)	8	133.17***	5.28
Error	189	28.04	28.04

**Table 3:** Analysis of variance for sum of ovarioles from both ovaries (two generations removed from selection as described in *Ovariole number *section of the Methods or Results). Comparison of selected *vs*. control lines *via *a 3-way, mixed model ANOVA. Linetype (selected vs. control lines), treatment (starved or not starved), block (28 hours or 32 hours starvation) are fixed main effects. Their interactions are also fixed. Line refers to the five replicate lines in each linetype; line is nested within linetype and is random, as are the interactions of line with block and treatment. * =*P *≤ 0.05; ** =*P *< 0.01; *** =*P *< 0.001

Source	Df	Estimated mean squares	Variance component
Line type (selected vs. control lines)	1	4965.13***	(fixed)
Treatment	1	545.31***	(fixed)
Block	1	1.71	(fixed)
Line type * treatment	1	47.04	(fixed)
Line type * block	1	220.83	(fixed)
Treatment * block	1	291.21*	(fixed)
Line type * treatment * block	1	7.26	(fixed)
Line (line type)	8	191.32	2.420
Line (line type) * treatment	8	18.58	0.000
Line (line type) * block	8	80.83	0.918
Line (line type) * treatment * block	8	53.29**	2.153
Error	560	20.99	20.992

**Table 4:** Ovariole number (sum of ovarioles from both ovaries) and wing length of daughters from unstarved mothers two generations removed from selection (as described in *Ovariole number *section of the Methods or Results). Selection for female starvation resistance did not result in an increase in linear body size, and linear body size is not correlated with ovariole number within lines.

Line type	Line number	Mean ovariole number ± std. error	Mean wing measurement (mm) ± std. error
control	1	31.80 ± 1.20	1.607 ± 0.008
control	2	36.60 ± 1.64	1.654 ± 0.008
control	3	32.10 ± 1.27	1.661 ± 0.010
control	4	34.20 ± 2.13	1.669 ± 0.008
control	5	35.60 ± 1.32	1.669 ± 0.007
selected	6	41.90 ± 0.85	1.615 ± 0.008
selected	7	39.70 ± 0.58	1.628 ± 0.009
selected	8	40.90 ± 1.22	1.698 ± 0.008
selected	9	44.40 ± 1.17	1.667 ± 0.009
selected	10	38.90 ± 1.16	1.682 ± 0.007

Maternal starvation increased the number of ovarioles in progeny of selected and control lines females. Two generations removed from selection, there was a significant effect of parental starvation resulting in increased ovariole number (starved vs. unstarved: 42.22 vs. 40.32; treatment; *P *< 0.001) as well as significant interactions between block and treatment, and line type (selected vs. control lines), treatment, and block (Table 3; *P *< 0.048 and 0.001, respectively). A similar result was seen when parental flies were taken directly from the selected and control lines (Table 1). Line type (selected vs. control) was again significant (control = 34.48, selected 42.17; *P *< 0.001), as was maternal starvation (starved vs. unstarved dams: 39.04 vs. 37.61; *P *< 0.002). There was evidence for genetic variation within selected and control lines(line [line type]) for ovariole number in flies whose dams were zero or one generation removed from selection (Tables 1 and 2, line (line type), *P *< 0.0001). There was no statistical support for genetic variation within selected or control lines in flies whose dams were two generations removed from selection (Table 3), but this is likely more reflective of the relatively large and significant line (line type) * treatment * block term in the denominator of the *F *test rather than reflective of the biology.

### Egg production

**Figure 2:**
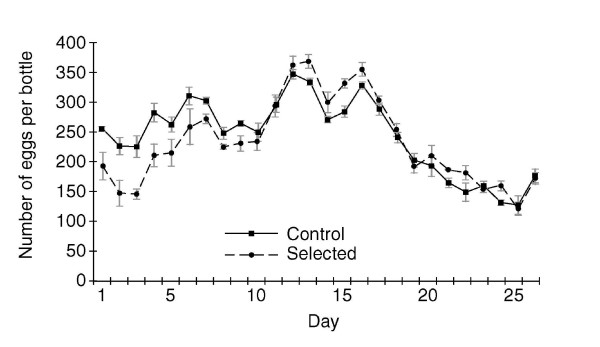
The daily fecundity schedule for the selected and control lines. The grand average per day was the overall mean number of eggs for selected lines and control lines per bottle with ten females in each bottle.

Figure 2 presents the daily line type grand average number of eggs for selected and control line females for each of the first 26 days of adult life. The total lifetime egg production per female was similar for selected and control line females (line type *P *= 0.1113; control, 6319.30; selected, 6037.80). There was no significant genetic variation among lines within selected or control lines (line [line type]; *P *= 0.19250). The following were the line means for control: 6058.0, 6245.0, 6326.0, 6383.5, 6584.0 and line means for selected: 5543.0, 5994.0, 6177.0, 6214.5, 6260.5). However, there was strong support for an effect of day (*P *< 0.001) and an interaction between day and line type (selected vs. control lines; *P *< 0.0001) on daily fecundity. Motivated by the interaction, a comparison of selected and control lines each day was made assuming an AR(1) process. There were statistically significant differences on days 1–6 (*P *< 0.001), when the selected lines were producing substantially fewer eggs than the unselected lines. After day 11, the selected line females tended to produce relatively more eggs for the remainder of the study, but this difference was not statistically significant.

### Wing length

There was no effect of selection on wing length when selected females were one generation removed from the selection regime (Table 4; *P *= 0.779). Further, there is no relationship between ovariole number and body size, either between selected and control lines (line type) or within each type of line (line [line type]). Across line types, the correlation between ovariole number and body size is not significant (*R *= 0.09776; *P *= 0.7882). Within line types, there is also no relationship between the two traits (control: *R *= -0.6385, *P *= 0.2462; selected: *R *= 0.6001, *P *= 0.2846). Results from non-parametric analyses are consistent with parametric analyses (data not shown).

### Starvation resistance

To assess the direct response to selection at the end of the present study, starvation resistance was assessed in the same manner used in the selection experiment except that starvation continued until all flies died. The grand mean female survival time under starvation was 98 (+/- 10) hours for the selected lines and 62 (+/- 15) hours for the control lines. These mean values are similar to those for selected and control line females at generation 27 [21] which was five generations before the present study began. Thus, the marked difference in starvation resistance between selected and control line females, was maintained throughout the present study.

## Discussion

Selection for increased adult female starvation resistance resulted in a correlated increase in the number of ovarioles. Similarly, maternal starvation resulted in an increase in offspring ovariole number. The present study was consistent in terms of an increase in ovariole number in selected females, irrespective of the assay generation or of the number of generations removed from the selection regime. Moreover, maternal starvation consistently resulted in an increase in progeny ovariole number in both control and selected line females. One might expect the effect of maternal starvation on ovariole number to be substantially greater in control females because 28 – 32 hours of starvation would be expected to have greater physiological impact on these females than on selected females. On the other hand, selected line females might have evolved to become more responsive to starvation conditions. Our study provides evidence for a consistent effect of starvation on ovariole number, which should motivate additional research on underlying mechanisms including the effect of duration of maternal starvation in the context of evolved starvation resistance.

The lines used for the present study were produced by selection only on female starvation resistance [22] because of the heterogeneity observed among starvation-selected lines when females and males were selected [19]. Both female and male starvation resistance evolved in response to selection on females [22]. Indirect male responses to selection have not been investigated in lines selected for female starvation resistance [22]. It is not clear what the likely correlated responses in males would be, but it might be the case that male reproductive performance paralleled the pattern of female egg production, showing a relative decrease at early ages in selected lines (Figure 2). Selection only on females might have contributed to the pattern of indirect responses in the present study. However, selection only on females probably does not explain the relationship between starvation resistance and reduced early age fecundity because a decrease in early age fecundity was previously observed to be a correlate of selection for starvation resistance when both sexes were employed in the selection regime [20].

The indirect responses to selection, increased ovariole number and decreased early age fecundity, were not a result of cryptic selection for an increase in linear body size, because there was no difference in wing length between selected and control lines. Wing length was chosen as an estimate of body size in preference to estimates of mass for two reasons: first, because differences in ovariole number could contribute to differences in mass; and second, because it is well established that laboratory selection for starvation resistance results in an accumulation of lipid in flies [18,19] and hence an increase in weight. Body size as estimated by wing size or thorax length is often correlated with development time [23-25]. Generally, slower development time was correlated with greater thorax or wing length and vice versa, but this relationship was not found in all studies. In any case, if there was any difference in development time between selected and control lines in the present study, then it was not manifest as a difference in a linear measure of body size (wing length).

The response to selection in the present study might conform to the Y model of resource allocation in which endogenous resources are allocated to either survival or reproduction [26]. When lines were selected for starvation resistance early in life, female flies responded by reducing early age reproduction in favor of survival under starvation conditions. Although the indirect responses of similar laboratory selection experiments using Drosophila are sometimes heterogeneous [3,27], our results were consistent with another selection experiments in which starvation resistance was genetically correlated with reduced early age fecundity [20]. Thus, in both studies it is plausible that the females selected for early age starvation resistance have shifted energy away from reproduction to survival under starvation conditions.

Energetic studies on selected and control lines of *D. melanogaster *are relevant to the relationship between reproduction and starvation resistance. Energy storage compounds have been measured in lines selected for starvation resistance and there is an increase in lipid and glycogen content [19-21,28,29]. Reduced reproduction does not arise from relatively low metabolic rate in the selected lines; there is little evidence that metabolic rate decreases in lines selected for starvation resistance [22,30]. One might expect a tradeoff between somatic energy storage and reduced energy in the reproductive system for egg production, but is not clear that energy for reproduction is limiting in the laboratory environment [31].

An alternative to an energetic explanation for the observations in the present study is derived from consideration of the role of molecular signaling in life history evolution [32]. The basic argument is that the ovary could be a source of signals that affect the soma independent of the role of the ovary as a sink for nutrients used for reproduction. As reviewed in Leroi (2001), [32], there is evidence for such signals in *Caenorhabditis elegans *and these signals require a functional insulin signaling pathway. The *D. melanogaster *insulin signaling pathway is conserved in that it shares common features with pathways in *C. elegans *and mammals [33]. The insulin signaling pathway could play a role in biochemical and life history clines documented for *D. melanogaster *[13]. However, a homozygous null mutation in the *D. melanogaster *insulin receptor substrate protein gene, which blocks normal insulin signaling, has little effect on ovariole number in an outbred wild type genetic background [34]. Nevertheless, signaling from the ovary could act as a mediator of egg production as an indirect response to selection for starvation resistance.

Greater numbers of ovarioles in the selected lines were correlated with reduced early age fecundity, but there was no correlation with overall fecundity. Earlier studies found a negative genetic correlation between ovariole number and early age reproductive maturation in a selection experiment for longevity and late age reproduction although there was no clear association between late life ovariole number and altered vitellogenic egg maturation [35,36]. Does the early age negative correlation imply that that there is a tradeoff between ovariole number and early age fecundity? Perhaps, but it will be necessary to establish a functional relationship between the traits to rigorously document whether a tradeoff exists [37]. Such a tradeoff may be relatively pronounced in the context of starvation resistance. Many studies of unperturbed natural populations have found a positive association between ovariole number and early fecundity [38-40]. Candidate genes associated with starvation resistance tend to be in developmental pathways responsible for cell fate [41]. These genes might exert pleiotropic effects during the pre-adult stages when ovarioles develop, whereby alleles conferring starvation resistance could cause an increase in ovariole number. Interestingly, an increase in the number of ovarioles would be expected to increase the total number of oocyte stem cells per ovary; and that could promote greater fecundity later in life assuming that a decline in stem cell number and function underlies the decline in late-age reproductive potential [42], as well as the lack of difference in total 26 day fecundity between selected and control lines in this study, despite the decrease in early fecundity in the selected lines. This could also explain the positive genetic correlation between longevity and ovariole number in lines selected for late life reproduction and extended longevity [36].

There is an alternate interpretation of the phenotypic manipulation experiments reported in the present study. In the absence of a suitable egg laying substrate during the time females were held in empty bottles for the starvation treatment, fertilized eggs might develop within the females. If such internal development resulted in a head start for these eggs; and if the first eggs laid after 28–32 hours of starvation comprised a large proportion of the progeny used for ovariole measurements, then it is possible that the relative maturity of these eggs could contribute to an increase in ovariole number. However, such developmental processes could not explain the increase in ovariole number in selected lines relative to control lines, because the difference in ovariole number persisted even in control lines two generations removed from the selective regime; and thus egg retention and accompanying accelerated maturation due to lack of oviposition substrates did not occur.

The clines in ovariole number within *D. melanogaster *[8-11] suggest that there is a relationship between a temperate environment and increased ovariole number. However ovariole number and starvation resistance are not known to positively covary in natural populations; a shallow cline in starvation resistance among Australian populations [43] appears to be negatively correlated with an Australian cline in ovariole number [11].

In general, there is a negative relationship between early age fecundity and stress resistance in selection experiments [37]. Moreover, phenotypic manipulation experiments have documented a negative relationship between egg production and starvation resistance [44,45]. Similarly, increased stress resistance, reduced early age reproduction, retarded vitellogenic oocyte maturation and increased ovariole number were all indirect responses to selection for late life reproduction and longevity [35,36,46-48]. In the present study, a greater number of ovarioles was associated with reduced early age fecundity and resistance to a specific stressor, starvation. A genetic correlation between female starvation resistance and increased ovariole number, and phenotypic correlation between maternal starvation and increased ovariole number, is documented for the first time in the present study. In the present study, reduced early age egg production is associated with increased ovariole number, as is relatively high later age egg production; but it is not clear that the larger number of ovarioles was necessary for the relatively elevated later age egg production in the selected lines. As a possible underlying mechanism for the relationship between ovariole number and egg production in the present study, the ovary might be able to modulate the distribution and allocation of endogenous energy storage compounds in relationship to life history traits. As summarized in Chippindale et al. (1993), [44], various studies have implicated the ovary and the neuroendocrine system as controlling factors in cellular lipid content and energy storage compound distribution in the body. In terms of life history traits and physiological tradeoffs, the number of ovarioles might correspond to the physiological impact of the ovary as a mediator of energy storage compound allocation between reproduction and the soma. Whether caused by energy compound shifts in the adult or pre-adult developmental pathways, the parallel phenotypic and genetic association between starvation resistance and ovariole number warrants further investigation.

## Conclusion

Selection for female starvation resistance resulted in an increase in ovariole number as a correlated response to selection as well as a decrease in early age fecundity, but total egg production was not affected by selection. Selection was associated with relatively low early age egg production; there was a negative correlation between starvation resistance and early age egg production and a negative correlation between ovariole number and early age egg production. Phenotypic manipulation by starvation resistance resulted in an increase in ovariole number in progeny. Starvation and ovariole number in progeny are apparently genetically and phenotypically linked, but the functional basis for this association is not yet understood.

## Methods

### Culture conditions

The standard rearing condition for all of the present study includes control of larval density by counting eggs, rearing at 21°C under constant illumination and holding of adults at 21°C. These conditions were used throughout the present study. For each experiment, assay, or morphological measurement, all selection and control lines were simultaneously evaluated. Standard half-pint bottles and 10 dram vials were used in all experiments and a standard cornmeal, molasses, yeast medium was used to culture the flies.

### Artificial selection

The present study was conducted from generations 32 to 40 of laboratory selection for female starvation resistance. The details of selection are described in Harshman and Schmid (1998), [22], but they will be summarized here. After approximately two years of laboratory culture, a base stock of *D. melanogaster *was subdivided into 10 lines. Five lines were selected for female starvation resistance (referred to as the Si lines) and five lines were maintained as controls (referred to as the W lines). When flies were 3–4 days post-eclosion, which allows time for sexual maturation and mating; females were separated from males by light ether anesthesia and allowed to recover overnight on food. Females were placed in empty bottles with a water-saturated plug at a standard density. After 24 hours of starvation conditions, before significant mortality occurred, females from the control lines were transferred to fresh vials with *Drosophila *food and added live yeast. The selected line females continued to be held without food until approximately 50% mortality and the survivors were transferred to fresh vials with *Drosophila *food and added live yeast. In general, there was no, or a few percent, mortality when control females were exposed to starvation for 24 hours and typically 40 – 60% mortality (50% on average) across generations, among selected line females in the selection regime. Following the regime established at the beginning of the selection experiment [22], after 3 days in vials following sublethal starvation (control lines) or starvation selection (selected lines), all females were transferred to fresh *Drosophila *food with added yeast in bottles at similar adult density for egg collection. Larval density was controlled by transfer of approximately 75 eggs to each vial used to produce the next generation. Control of larval density by counting eggs was employed throughout the selection experiment and for all experiments in the present study.

### Ovariole number

The method for ovariole number determination was described in Carlson et al. (1998 [35]). Females for dissection were reared from multiple vials and randomized across vials. Randomized adult females were held for 5–7 days after eclosion with an equal number of males in vials at 21°C prior to being frozen. Females were frozen and held at -20°C until they were dissected for ovariole number determination. Typically, ovariole numbers were determined from these females within 30 days after they were frozen. Only a few females were thawed at any one time (typically 5 or less) to avoid tissue deterioration after the freeze-thaw process. Each female was placed in a drop of *Drosophila *Ringers solution (7.5 g NaCl, 0.35 g KCl. 0.21 g CaCl_2 _per liter of H_2_O) for dissection. The tip of the abdomen was pulled away in a posterior direction while the remainder of the body was held by a second pair of forceps. The ovarioles were gently separated from each other using tungsten needles. All ovarioles in both ovaries were counted in each female.

Three similar experiments were conducted on ovariole number. In all three experiments, ovariole numbers were determined in females from all selected and control lines. In two of the studies, a phenotypic perturbation (maternal starvation) was included in the comparison of selected and control line females. Studies were conducted on flies zero, one, or two generations removed from selection. The motivation for using flies variable numbers of generations removed from selection was to be able to determine if the response to the starvation as a phenotypic manipulation, or the magnitude of the ovary number indirect response to selection, was related to generational proximity to lengthy starvation as the selective agent in the selection experiment. Moreover, it provided for a high degree of repetition of the tests of relationship between starvation selection, maternal starvation and ovariole number.

For zero generations removed from selection, females used as dams in the experiments were taken directly from the starvation lines without intervening generations of rearing under control line conditions. Selected and control line parental females were either exposed to 28 hours of starvation, or not starved, prior to egg collection for the next generation which provided the daughters used for ovariole number determinations. For this experiment, ovarioles from both ovaries were counted from 10 females per line. For one generation removed from selection, the dams of the daughters for ovariole number determination were taken from lines in which selection had been relaxed for one generation. All of the ovarioles from both ovaries were counted from 20 females for each line. For two generations removed from selection, the dams of the daughters for ovariole number determination were taken from lines in which selection had been relaxed for two generations. This experiment included a comparison of ovariole number after maternal starvation treatment versus no starvation; half of the females from each selected and control line were starved and half were held without starvation. At the beginning of the experiment, females and males were held at 21°C for 5–7 days. Thereafter, females were separated from males and held at 21°C at a density of 10 per empty vial with a water-saturated cotton plug for 28 hours for parental starvation in one case and 32 hours of starvation in a second case. The longer period of time without food, 32 hours, was nearly the maximum duration of starvation without significant mortality. The motivation for using the longest possible sub-lethal starvation period in one experiment was to investigate the maximal physiological impact of maternal starvation on offspring ovariole number. After the starvation period, all females were transferred to fresh food for egg collection. As always, a standard number of eggs (75 per vial) from starved and unstarved, selected and control line females was collected and transferred to each vial used to produce offspring to be assayed. After eclosion, males and females from the next generation were held together on fresh food for 5–7 days. After this time females were frozen for ovariole number determination. Ovariole numbers were counted for both ovaries from 15 females per line. Analysis was performed on the sum of the ovarioles from both ovaries.

### Early age egg production

The focus of this assay was to determine the pattern of early age egg production. Flies from the lines selected for female starvation resistance, and control lines, were used to assess daily egg production for 26 days post-eclosion. Two generations of controlled density culture at 21°C were conducted without starvation prior to collecting flies for the egg production study.

The egg production assays employed cut bottles with *Drosophila *food. The bottles were cut completely through around the circumference approximately 2.5 cm above the bottom of each bottle. During the process of preparing *Drosophila *food, molten medium was poured almost up to the cut that separates the bottom and top half of the bottles. When the food cooled, the top of the bottle was attached to the lower portion of the bottle with masking tape. Two bottles were used for each of the 10 lines (5 starvation selected lines and 5 control lines). Each bottle contained 10 females and 10 males, and the flies were 4 days old at the beginning of the study. Every day for 26 days, the flies in the bottles were transferred to new cut bottles with *Drosophila *medium. The previous day's bottles were disassembled and the disk of medium in the lower portion of the bottle was removed and placed on a piece of wax paper (the egged surface side up) in a box. The *Drosophila *food disks, which contained eggs produce during a 24 hour period, were frozen at -20°C to be thawed later and eggs counted.

### Wing length

Wings were measured to appraise whether fecundity and ovariole number differences between selected and control lines was correlated with changes in linear body size. The relevance to the present study is that linear body size has often been found to be correlated with fecundity. A genetic correlation between ovariole number and body size was not found in an earlier study [49], but that did not preclude an increase in linear body size as a response to selection for starvation resistance. A genetic correlation between ovariole number and starvation resistance in our study could have been caused by a difference in linear body size between selected and control line females.

The measurements were taken under 20× magnification using an ocular micrometer. Wing length was measured as the linear distance from the intersection of the anterior crossvein to the wing margin at the distal end of the third longitudinal vein. Both wings were removed from each female on day 4 post eclosion and the wings were positioned under a coverslip on a glass slide. Twenty pairs of wings were measured for each selected and control line.

### Starvation resistance

At the end of the selection experiment, starvation resistance was determined for selected and control lines. At generation 40, selection was relaxed for two generations before starvation resistance was determined in the selected and control lines before the selection experiment was terminated. Starvation resistance was determined using replicate bottles for each selected and control line. For starvation assays, females were placed in empty bottles with water-saturated plugs. This assay allowed for comparison to the direct response to selection in these lines assessed in an earlier generation [21].

### Statistical analysis

The general form of the statistical analysis used in the present study was to test for grand mean line type (selected and control lines) differences, and treatment differences, using an error term that included the variation between replicate lines of the same type. Ovariole number and wing length were statistically analyzed by ANOVA using the GLM procedure of the SAS package, and variance components estimated using PROC VARCOMP.

Daily egg production for 26 days was analyzed as repeated measures. Specifically, the autoregressive 1 process, AR(1), provided a better fit to the data than other covariance structures tested including compound symmetry. The data were analyzed as a hierarchical ANOVA using an error term that included the variation between replicate lines of the same type (two replicate bottles were set up for each line). The MIXED procedure of the SAS package was used for the analysis. The data was analyzed untransformed or square root transformed; the results of the statistical analyses were essentially the same and only the analysis of the square root transformed data is reported in the present study.

## Abbreviations

ANOVA = analysis of variance, SE = standard error, GLM = general linear model, df = degrees of freedom

## Authors' contributions

MW and LH conceived of the project. MW conducted most of the statistical analyses, made

major contributions to writing the manuscript and revisions, and figures. US collected the data in the LH laboratory. US entered the data into spreadsheets and provided summaries of the data for figures. US generated a rough draft of one figure. LH designed the experiments, wrote the first draft of the manuscript and made major contributions to manuscript revisions.
